# Cocaine in Venereal Surgery

**Published:** 1887-09

**Authors:** A. A. Lyon

**Affiliations:** Tyler, Texas, Ex-Pres. La. State Medical Association


### COCAINE IN VENEREAL SURGERY.

*By A. A. Lyon, M. D., Tyler, Texas, Ex-Pres. La. State Medical Association.*

[For Daniel's Texas Medical Journal.]

My attention was recently attracted by an article from the pen of D. Berry, M. D., of San Antonio, which appeared in the July number of this Journal, on the subject of "Muriate of Cocaine as a local anesthetic in the cautery of venereal sores." I simply desire to add my testimony to his, touching the efficacy of this agent when properly applied. For the past eighteen months it has been my unvarying practice to resort to cocaine as a preliminary or a measure preparatory to the cuuterization by nitric acid of all venereal sores requiring it, and especially those purely chancroidal. When thoroughly and sufficiently applied, I have found it to prove a perfect anæsthetic, no pain whatever being complained of. In some instances, however, the pain has been slight, due I think to an insufficiency either as to quantity of the cocaine used or of the time intervening between its application and that of the acid. My rule of late has been to apply freely to the sore and the tissue immediately surrounding it, with a camel's hair pencil, or what is perhaps preferable, drop from a pipette a few minims of a four per cent. solution of the cocaine muriate directly upon the affected part.

Immediately following this, I saturate a pledget of absorbent cotton, with the same solution, and lay it upon the sore, and allow it to remain from four to six minutes. By these means you will scarcely fail to produce complete local anæsthesia and the cotton being removed, the fuming acid is applied without any pain at all, or pain so slight, as scarcely to be appreciated. At a final application I replace the cotton pledget or a fresh one similarly saturated. From my experience this method has proved most satisfactory, and I feel confident in recommending it in all such cases.

From the well established virtues of muriate of cocaine as a local anæsthetic, especially in surgery involving the mucous membranes, it would seem most natural that the profession would be led to try it in this particular class of cases, and I suspect that many others besides Dr. Berry and myself have practiced it, time and again, but until Dr. B's report I had never seen it alluded to in any medical literature that has ever come under my observation, and for sometime past have intended to make public my experience, as above detailed.

I will state further, that encouraged by this practice in the case of chancroids I determined to test its merits, as a helper in lancing buboes,—those sequent concomitants of the preceeding chancroid. In this too I have a most favorable report to make. As every well informed physician knows the application of muriate of cocaine to the cutaneous surface proper, gives negative results so far as anæsthesia is concerned, or if any effect at all is produced it is so slight as not to be recognized. It is however different when sub-cutaneously injected. Prior to opening a bubo therefore I have been accustomed to inject with the ordinary hypodermic syringe, from ten to thirty minims of the four per cent solution into the skin and the tissue immediately underlying, a few minutes before the incision is made. Of course the quantity of the solution to be used and the depth it is inserted depend upon the size of the bubo, and the extent of the proposed incision, which every operator must determine for himself. Ordinarily my plan is as follows: About twenty minims of the solution (four per cent) is drawn into a syringe, armed with a long sharp needle. The needle is then inserted obliquely from above downward, in the direction of the axis of the bubo—the point being held just beneath the surface of the skin. The course of the needle should be in the exact line of the intended cut and the linear extent traversed, i. e. the distance from the point of puncture to the end of the needle *in situ* prior to its withdrawal, should exceed, a little, the length of the incision to be made. The puncture being complete, let the needle be slowly and steadily withdrawn, and the solution as gradually and uniformly injected along the entire tract, the last minim being deposited just as the needle is removed. In this first injection about half the contents of the syringe should be employed. A second puncture is then made precisely in the same manner from the opposite direction (i. e. obliquely from below upwards), but more deeply, so as to include the subcutaneous tissue as nearly as possible *immediately* underlying the skin already treated. A third puncture still deeper might be made if the abscess is very deep seated, but I have never had occasion to employ but the two as just described. After an interval of five or six minutes let the incision be made directly, if possible, down through the tracks of the needle, which of course will involve all the tissue anæsthetized. Up on these conditions a sufficiently deep cut may be made to relieve the bubo, without any pain whatever, or the slightest inconvenience to the patient. One instance I recall in which a bubonic abscess was opened, without even the knowledge of the party, he asking me, after the operation was complete, "if I had opened it yet," and expressed surprise to know that everything was over.

I have been somewhat minute in describing the manner of inserting the cocaine, because upon great care in this particular, depends in my judgment, the painlessness of the proceedure, and even with the greatest care there may sometimes be slight pain,—but very slight. The solution injected carefully into the tissue, without including all that portion involved by the incision will not be painless, as one will readily understand, hence the particularity recommended in the preparatory part of the operation. Upon success in this respect the end we seek can alone be obtained. The insertion of the needle occasions no greater pain than the ordinary use, in any other portion of the body; at least I have so found it.

Now, these are minor points we may say, in minor surgery—but they are practical points, and are well worth the favorable consideration of medical men, dealing daily with just such cases as have been described. By attention to these details we may save in the aggregate, a vast deal of temporary suffering, to thousands of our unfortunate fellow men whose rambles into forbidden paths have gotten them into trouble, while we ourselves in our back offices, are spared the old familiar, yet harrowing (?) scene of unhappy grimaces and gyrations, oaths and perspirations that so generaliy obtained under the old regime.

